# Evaluation of the Zoonotic Potential of Transmissible Mink Encephalopathy

**DOI:** 10.3390/pathogens2030520

**Published:** 2013-07-30

**Authors:** Emmanuel E. Comoy, Jacqueline Mikol, Marie-Madeleine Ruchoux, Valérie Durand, Sophie Luccantoni-Freire, Capucine Dehen, Evelyne Correia, Cristina Casalone, Juergen A. Richt, Justin J. Greenlee, Juan Maria Torres, Paul Brown, Jean-Philippe Deslys

**Affiliations:** 1 CEA, Institute of Emerging Diseases and Innovative Therapies (iMETI), Division of Prions and Related Diseases (SEPIA), Route du Panorama, BP6, 92265 Fontenay-aux-Roses, France; E-Mails: jacqueline.mikol@wanadoo.fr (J.M.); mruchoux@yahoo.fr (M-M.R.); valerie.durand@cea.fr (V.D.); sophie.luccantoni@cea.fr (S.L.); capucine.dehen@cea.fr (C.D.); evelyne.correia@cea.fr (E.C.); paulwbrown@comcast.net (P.B.); jpdeslys@cea.fr (J-P.D.); 2 Istituto Zooprofilattico Sperimentale del Piemonte, Via Bologna 148, 10154 Torino, Italy; E-Mail: cristina.casalone@izsto.it (C.C.); 3 Kansas State University, College of Veterinary Medicine, K224B Mosier Hall, Manhattan, Kansas 66506-5601 USA; E-Mail: jricht@vet.k-state.edu; 4 National Animal Disease Center, USDA, Agricultural Research Service, 1920 Dayton Ave, Ames, Iowa 50010 USA; E-Mail: justin.greenlee@ars.usda.gov (J.J.G.); 5 Instituto Nacional de Investigacion y Tecnologia Agraria y Alimentaria, Madrid, Spain; E-mail: jmtorres@inia.es

**Keywords:** primate, prion, transgenic mice, TME, cattle, raccoon, zoonotic potential

## Abstract

Successful transmission of Transmissible Mink Encephalopathy (TME) to cattle supports the bovine hypothesis for the still controversial origin of TME outbreaks. Human and primate susceptibility to classical Bovine Spongiform Encephalopathy (c-BSE) and the transmissibility of L-type BSE to macaques indicate a low cattle-to-primate species barrier. We therefore evaluated the zoonotic potential of cattle-adapted TME. In less than two years, this strain induced in cynomolgus macaques a neurological disease similar to L-BSE but distinct from c-BSE. TME derived from another donor species (raccoon) induced a similar disease with even shorter incubation periods. L-BSE and cattle-adapted TME were also transmissible to transgenic mice expressing human prion protein (PrP). Secondary transmissions to transgenic mice expressing bovine PrP maintained the features of the three tested bovine strains (cattle TME, c-BSE and L-BSE) regardless of intermediate host. Thus, TME is the third animal prion strain transmissible to both macaques and humanized transgenic mice, suggesting zoonotic potentials that should be considered in the risk analysis of animal prion diseases for human health. Moreover, the similarities between TME and L-BSE are highly suggestive of a link between these strains, and therefore the possible presence of L-BSE for many decades prior to its identification in USA and Europe.

## 1. Introduction

Transmissible Mink Encephalopathy (TME) is a rare prion disease affecting ranch-reared mink that was reported in four isolated outbreaks in the USA in 1947, 1961, 1963 and 1985 [1], and in several other outbreaks in Canada, East Germany, Finland and the former USSR during the same time period, with prevalence rates as high as 100% and an estimated incubation period of 6 months [2]. Epidemiological studies suggested that each outbreak was due to dietary infection. 

Several experimental exposures of mink to ruminant prions were performed to identify the exact origin of TME. Low efficiency and rate of transmission were observed after inoculation of mink with sheep scrapie [3] and elk-derived Chronic Wasting Disease (CWD) [4] isolates with an incubation time of 2–3 years, while a 100% success rate of transmission was obtained within 12 months post-exposure to classical Bovine Spongiform Encephalopathy (c-BSE) [5]. However, in all cases, the resulting diseases differed from TME. Conversely, TME was experimentally transmitted to cattle [6,7] inducing a prion disease distinct from c-BSE within 16 to 28 months. Experimental transmissions to conventional and transgenic rodent models suggested similarities between TME and L-BSE [8,9], an atypical cattle prion strain that was incidentally identified several years ago in aged cattle through systematic testing within the framework of the European BSE epizootic [10]. It was speculated that sporadic atypical cattle BSE (H- and/or L- type) might be at the origin of c-BSE [11,12]. These observations support the hypothesis of a bovine origin to TME.

Currently, classical BSE is the only animal transmissible spongiform encephalopathy (TSE) considered as a zoonotic disease, since it induces a variant of Creutzfeldt-Jakob disease (CJD) in humans [13,14,15]. We, and others, demonstrated that the cynomolgus macaque, previously used to demonstrate the transmissibility of human prion diseases [16], constitutes a relevant experimental model to assess the BSE risk for humans [14,17,18,19,20]. The same species was also susceptible to L-BSE [21,22], developing a disease distinct from c-BSE. Taken together, these results suggested a low cattle-to-primate species barrier and raised questions about the zoonotic potential of different bovine prion strains. 

We chose to assess the risk for human health linked to TME-related prion strains by evaluating the transmissibility of cattle-adapted TME in this cynomolgus macaque model, in comparison to raccoon TME as a non-ruminant source of the same prion strain. In parallel, we used transgenic mice overexpressing human or bovine prion protein (PrP) to assess the relevance of our results for human situation.

## 2. Results and Discussion

### 2.1. Transmission of Cattle-Adapted TME in Experimental Models

A primate intracerebrally inoculated with the equivalent of 40 mg of a TME-infected cattle brain (second passage) developed the first neurological signs of disease after less than twenty months of incubation (Table 1). It first showed slowness and weak tremors amplifying with time. Clinical signs then evolved with ataxia, hypermetria, poor vision, and apparent cognitive impairment. Appetite remained normal during the entire 3.5 months clinical period (limited weight loss) and no behavioral changes were noticed (total survival period 23 months). The presence of cerebral spongiosis and protease-resistant prion protein (PrPres) deposition (detailed hereafter) confirmed the presence of prion disease. When another, non-ruminant, source of TME was injected, disease occurred with a similar period of survival (Table 1). 

**Table 1:** Survival (incubation and clinical duration) in months of individual cynomolgus macaques exposed to different prion strains.

Dose	Cattle TME	Raccoon TME	L-BSE	c-BSE
100 mg				40 *(38 / 2)*
40 mg	23 *(19.5 / 3.5)*	18 *(11.5 / 6.5)*		
25 mg			26 *(21.5 / 4.5)*	
2.5 mg			25 *(20 / 5)*	
0.5 mg				57 *(55 / 2)* 93 *(85 / 8)*

**Figure 1:**
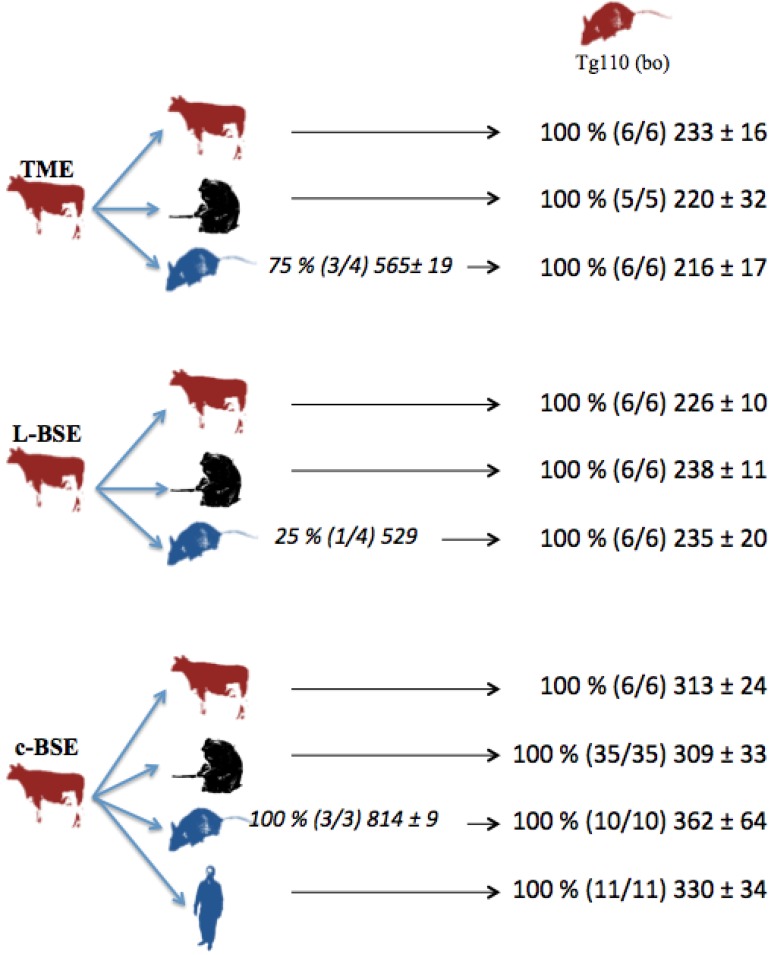
Transmission studies of bovine prion strains to transgenic mice overexpressing human (tg650) or bovine (tg110) PrP. Tg650 mice (colored in blue) were intracerebrally inoculated with 20 µl of 10 % brain homogenate from cattle infected with adapted TME, L-BSE or c-BSE strains. Tg110 mice (colored in red) were inoculated directly with the same cattle inocula, or with brains from macaques or tg650 mice previously exposed to those cattle inocula. vCJD inoculum was injected as controls. Transmission results are expressed as rates of transmission (%), number of recipient mice (in brackets), and mean ± standard deviation of their incubation periods.

In parallel, several but not all the transgenic mice overexpressing human (Met/Met) PrP (tg650 mice) intracerebrally inoculated with cattle-adapted TME inoculum exhibited cerebral PrPres: partial transmission (75 %) occurred in humanized mice that died after about 18 months of incubation (Figure 1).

### 2.2. Transmission of other cattle prion strains

From these results, cattle-adapted TME represents the third cattle prion strain (together with c-BSE and L-BSE) experimentally demonstrated to be transmissible to non-human primates. We confirmed in this study the previously described transmissibility of both L-BSE and c-BSE in both experimental primates [14,21] and transgenic [23,24] models. 

In the primate model, exposure to L-BSE-infected cattle brain induced a clinical picture with incubation time and duration of illness that are similar to those observed after exposure to cattle-adapted TME, even after exposure to as little as 2.5 mg of brain tissue (Table 1). Conversely, c-BSE infected primates developed a different clinical picture, as previously described [14,21], with longer incubation periods even when they were exposed to 100 mg of brain tissue.

A comparison of incubation periods confirmed and magnified the higher virulence of L-BSE for macaque compared to c-BSE, which we had previously observed [21]. Moreover, since incubation periods classically increase with the dilution of initial infectious amount in experimental prion diseases, the similarity of incubation durations for primates exposed to either 25 or 2.5 mg of L-BSE-infected brain is in favor of a substantial amount of infectivity in the brains of cattle infected with the L-BSE prion strain.

In the model of transgenic mice overexpressing human (Met/Met) PrP (tg650 mice), an incomplete transmission rate of 25% of L-BSE was observed after an incubation period of similar duration (18 months) to those from cattle TME-exposed animals, while a 100% transmission rate was observed with c-BSE, but with longer incubations (animals were euthanized 27 months post inoculation, corresponding to the lifespan of these animals in our facilities). These observations are consistent with the results obtained with L-BSE strain by Beringue *et al*., in this transgenic model [24] and by Kong in another humanized mouse model [25], suggesting a less efficient transmission of L-BSE and TME than c-BSE in this transgenic model, but with a shorter evolution when it occurs.

The overexpression of PrP in transgenic mice is often criticized as an element helping to force the way through the species barrier and extrapolation of our results in this model to the human situation should be taken with caution, since transgenic mice expressing physiological levels of human PrP are resistant to L-BSE [26]. Nevertheless, it must be noted that these ‘physiologic’ mice are also resistant to c-BSE, impairing its relevance for assessing the zoonotic potential of animal prion strains. In any case, an efficient transmission of these prion strains to primate, possibly in the presence of a weak cattle-to-primate species barrier, may be extrapolated from our results in the macaque model, which is strengthened by the transgenic model and the current absence of any argument for a zoonotic potential of prion strains derived from other ruminants (ovine or caprine classical or atypical scrapie, wild ruminant CWD). 

### 2.3. Comparative Pathologies of the Diseases Induced by the Different Cattle Prions

**Figure 2:**
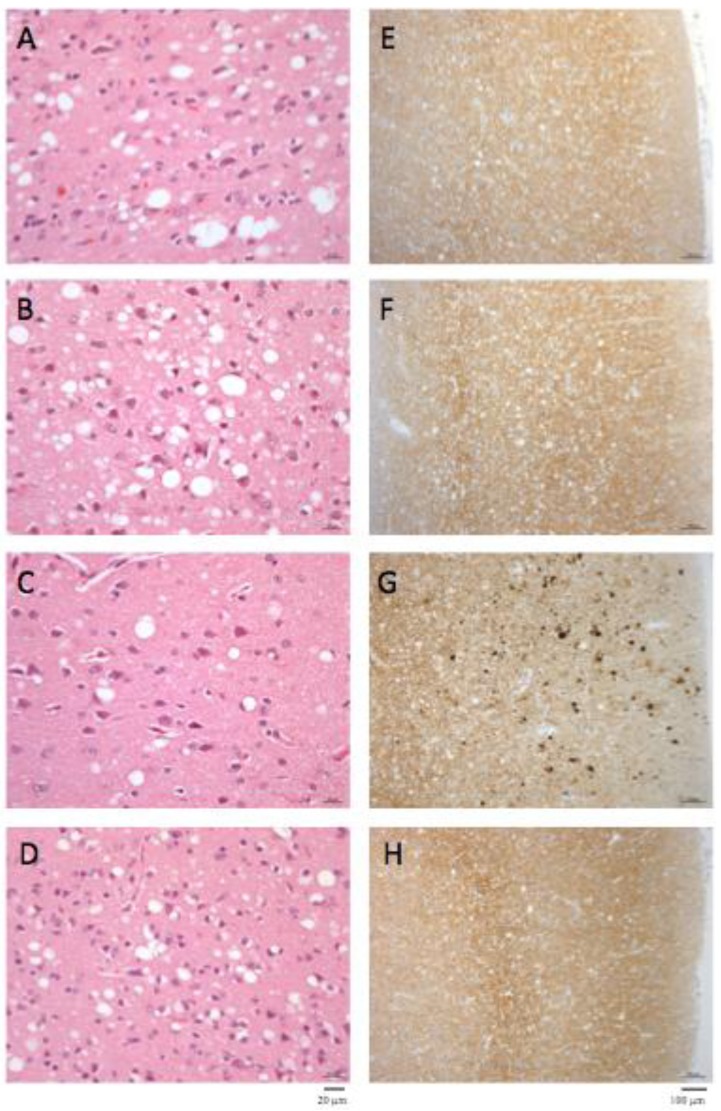
Histopathology and PrPres immunostaining. Spongiosis (A–D) and PrPres deposition (E–H) in frontal cortex in primates infected with cattle-adapted TME (A, E), L-BSE (B, F), classical BSE (C, G) or raccoon TME (D, H) (original magnification x200 for spongiosis and x400 for PrPres staining). Immunostaining of PrPres was performed with 3F4 monoclonal anti-PrP antibody after proteinase K treatment as previously described [21]. No staining was observed in the brain of control healthy primates (data not shown) under these conditions.

**Figure 3:**
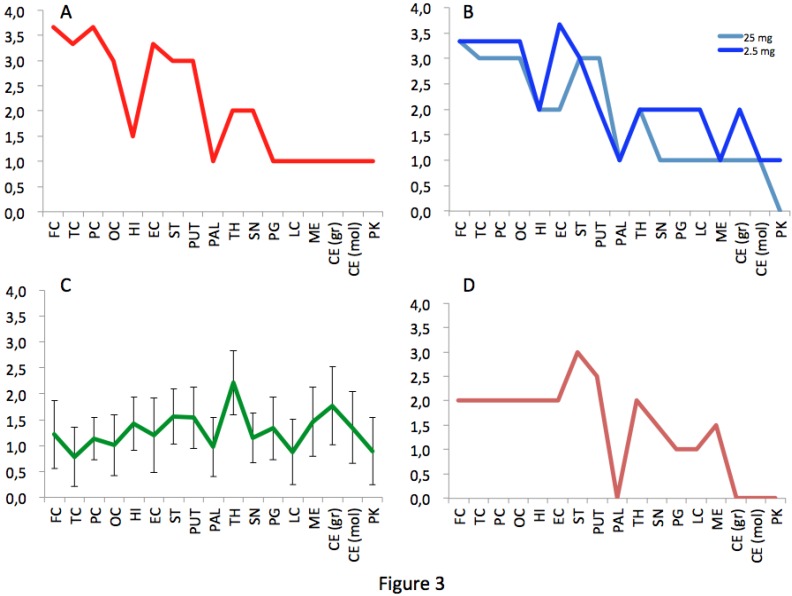
Spongiosis profiles in infected primates. Lesional profiles (based on spongiosis) in primates exposed to cattle-adapted TME (A) L-BSE (B), c-BSE (C) and raccoon TME (D) were defined according to the scoring and areas described by Parchi et al. [27]. Spongiosis profile of c-BSE primates is depicted as the mean among 5 primates exposed to c-BSE. Frontal Cortex (FC) ,Temporal Cortex (TC), Parietal Cortex (PC), Occipital Cortex (OC), Hippocampus (HI), Entorhinal Cortex (EC),Striatum (ST), Putamen (PUT), Pallidum (PAL), Thalamus (TH), Substantia Nigra (SN), Periventricular Gray (PG), Locus coeruleus (LC), Medulla (ME), Cerebellum (granules) (CB), Cerebellum (molecular layer) (CB), Purkinje cells (PK).

The macaque inoculated with cattle-adapted TME showed widespread cortical spongiosis similar to that in both primates exposed to L-BSE (Figure 2). The spongiosis profile for these three animals was superimposable, with less pronounced lesions in the medulla and cerebellum in cattle TME-infected animal than in L-BSE animals (Figure 3). In the c-BSE-inoculated macaques, spongiosis profiles were different, with more discrete cortical spongiosis and lesions mainly affecting the thalamus, medulla oblongata and cerebellum. When we used a non-cattle (raccoon) TME source, a similar spongiosis profile was observed but with slight modifications (cortical lesions were less pronounced and pallidum and cerebellum were virtually spared).

Primates inoculated with L-BSE or cattle TME exhibited a similar diffuse laminar synaptic pattern of PrPres depositions (either fine and sandy or roughly granular) but no evidence of plaques, even when stained with thioflavine T (data not shown), whereas c-BSE-infected animals had weak diffuse synaptic labeling but multiple intensely-stained PrPres aggregates and characteristic plaques [21]. 

### 2.3. PrPres Detection: Strain Discrimination by Proteinase K Sensitivity and Antibody Reactivity

We previously demonstrated that the technique that we developed for typing and classifying prion strains in small ruminants might also be used to discriminate L-BSE from c-BSE in experimentally infected macaques [21]. Briefly, this technique is based on the strain-dependent threshold of removal of the octapeptides under controlled conditions of proteolysis, in which this N-terminal region is highly resistant to proteolysis for scrapie and sporadic CJD prions, but weakly resistant for c-BSE and undetectable for L-BSE.

**Figure 4:**
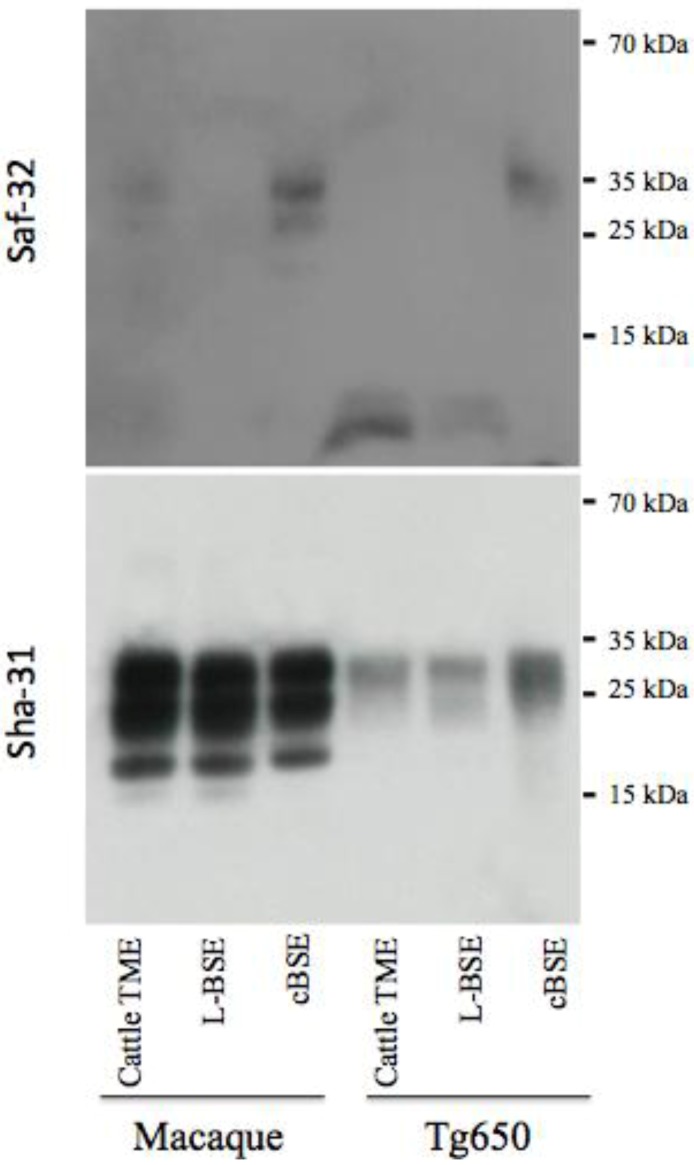
Electrophoretic analysis and differential sensitivity to proteolysis of PrPres in various experimental prion diseases of primates and transgenic mice overexpressing human PrP. PrPres from brain homogenates (primates or transgenic mice Tg650 overexpressing human PrP experimentally infected with cattle-adapted TME, L-BSE or c-BSE) were purified with high concentrations of proteinase K, and detected with monoclonal antibodies recognizing the octapeptide region (Saf-32) or the core (Sha-31) of the protein. The membrane blotted with Saf-32 was overexposed compared to the membrane blotted with Sha-31.

Under these experimental conditions, PrPres in both primates and Tg650 exposed to cattle-adapted TME behaved like PrPres derived from corresponding animals infected with L-BSE [21] (Figure 4). A 19 kDa non-glycosylated band was observed with the anti-core antibody Sha-31, with an equal distribution between mono- and diglycosylated bands. With the anti-octapeptide antibody Saf-32, almost no immunoreactivity was detectable for these animals. In parallel, c-BSE infected animals exhibited the expected features, including a 20 kDa non glycosylated band with Sha-31, predominance of diglycosylated band and a weak immunoreactivity with Saf-32 (only observable when overexposing the membrane), suggesting that c-BSE related PrPres is more resistant to proteolysis than L-BSE- or TME-related PrPres (the macaque infected with raccoon TME exhibited glycophoretic profiles and resistance to proteolysis resistance similar to the macaque infected with cattle TME, data not shown). We previously described the biochemical similarities between PrPres derived from L-BSE infected macaque and cortical MM2 sporadic CJD: those observations suggest a link between these two uncommon prion phenotypes in a primate model (it is to note that such a link has not been observed in other models less relevant from the human situation as hamsters or transgenic mice overexpressing ovine PrP [28]). We speculate that a group of related animal prion strains (L-BSE, c-BSE and TME) would have a zoonotic potential and lead to prion diseases in humans with a type 2 PrPres molecular signature (and more specifically type 2B for vCJD).

Strain signatures were also assessed in bioassays in transgenic mice overexpressing bovine PrP (tg110). Those mice were intracerebrally inoculated with cattle-adapted TME, L-BSE or c-BSE isolates issued from cattle, macaque or tg650 recipients (vCJD samples were also included as controls) (Figure 1). All the inoculated mice developed a TSE, with similar incubation periods whatever the source (cattle, primate, Tg650 mice or human), but related to the original prion strain: mean periods ranged from 216 to 233 days for cattle-adapted TME and from 226 to 238 days for L-BSE, while c-BSE led to longer incubation periods ranging from 309 to 362 days. 

**Figure 5:**
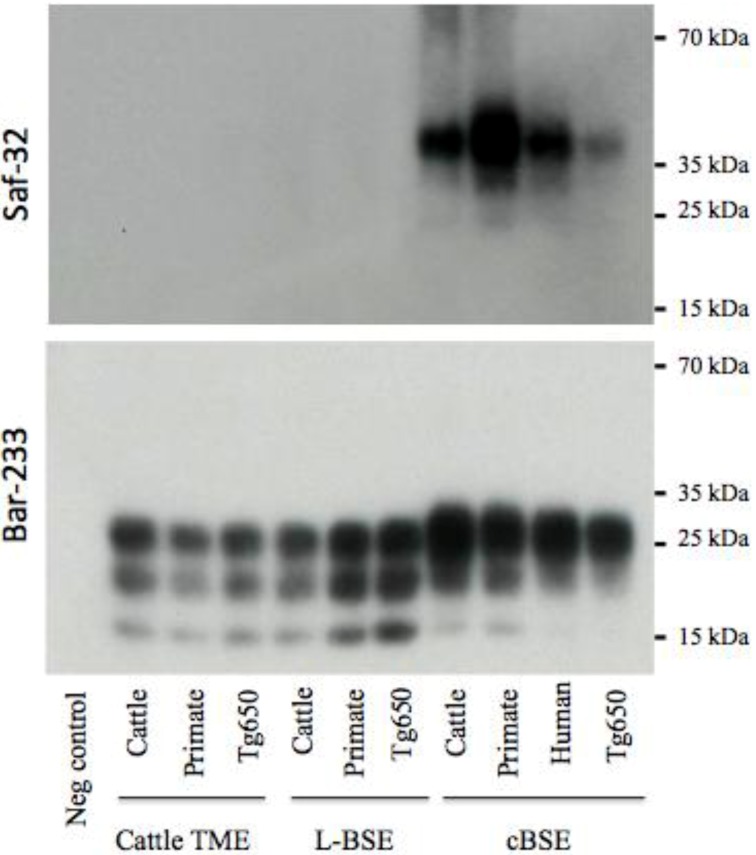
Electrophoretic analysis and differential sensitivity to proteolysis of PrPres in various experimental prion diseases of transgenic mice overexpressing bovine PrP. Transgenic mice Tg110 overexpressing bovine PrP were inoculated with brain tissue from cattle, primate, or Tg650 mice experimentally infected with cattle-adapted TME, L-BSE or c-BSE, or brain tissue from a vCJD patient. The brains were homogenized and PrPres was purified with high concentrations of proteinase K, and detected with monoclonal antibodies that recognize either the octapeptide region (Saf-32) or the core (Bar-233) of the protein. The membrane blotted with Saf-32 was overexposed compared to the membrane blotted with Bar-233.

This biochemical strain typing protocol was adapted (preferential use of Bar-233 antibody for protein core detection) and applied to Tg110 mice (Figure 5). The respective features (size of non glycosylated band, proportion of glycosylated forms, resistance of octapeptide regions to proteolysis) that were observed in primates and Tg650 mice for each original prion strain (cattle TME, L-BSE or c-BSE) were also observed in this model, regardless of the host from which the prion originated.

## 3. Experimental Section

### 3.1. Ethics Statement

Primates and mice were housed and handled in accordance with the European Directive 2010/63 related to animal protection and welfare in research, under the constant internal surveillance of veterinarians. Animals were handled under anesthesia to limit stress, and euthanasia was performed for ethical reasons when animals lost autonomy. 

### 3.2. Experimental Animals

Captive-bred 2-5 year-old male cynomolgus macaques (*Macaca fascicularis*) were provided by Noveprim (Mauritius), checked for the absence of common primate pathogens before importation, and handled in accordance to national guidelines. Transgenic mice overexpressing human (tg650 [23]) or bovine (tg110 [29]) PrP were internally bred at CEA (Fontenay-aux-Roses, France). Animals housed in level-3 animal care facilities (agreement numbers A 92-032-02 for animal care facilities, 92-189 for animal experimentation) were regularly examined at least once a week.

### 3.3. Experimental Inoculations

The TME inocula were derived from a second passage in cattle (#A263, [7]) or the first passage from mink to raccoon (#R5-6, [30]). The L-BSE inoculum (mix of brainstem and thalamus) was derived from an asymptomatic 15 year-old Italian Piemontese cow (#1088, [10]), and the c-BSE inocula were derived from infected UK cattle. Macaques and mice were intracerebrally (i.c.) inoculated with 1% or 10% brain homogenates in a 5% glucose solution. 

### 3.4. Neuropathology and Immunohistochemistry

Tissues were fixed in formalin 4% for histological examination. Neuropathology and immunohistochemical detection of protease-resistant prion protein (PrPres) were performed on brain sections as previously described [21]. 

### 3.4. PrPres Analysis

PrP was purified according to the TeSeE purification protocol (Bio-Rad), in adapted conditions of proteolysis for strain discrimination as previously described [21], using Bar-233, Sha-31 or Saf-32 antibodies. 

## 4. Conclusions

We have shown that cattle-adapted TME is the third cattle prion strain (joining classical and L-type BSE) to be transmissible both to non-human primates and transgenic mice overexpressing human PrP. However, the successful transmission of raccoon TME to primate, inducing a disease with similar features as cattle TME, extends this notion to TME-related strains independent of host origin. Pathological, biochemical and bioassay investigations converged to demonstrate the similarity between cattle-adapted TME and L-BSE. Together with previous experiments performed in ovinized and bovinized transgenic mice and hamsters [8,9] indicating similarities between TME and L-BSE, the data support the hypothesis that L-BSE could be the origin of the TME outbreaks in North America and Europe during the mid-1900s. The corollary of this notion is the longstanding existence of atypical bovine prion cases in those countries during the same period, if not earlier. Although the risk of L-BSE for public health must be further assessed through studies using the oral route of exposure before drawing definitive conclusions, these data underline the importance of a potential zoonotic risk of L-BSE in the management of consumer protection, particularly in the context of the current relaxation of European policy with respect to BSE. 
